# Sensitivity of contrast-enhanced transthoracic echocardiography for the detection of residual shunts after percutaneous patent foramen ovale closure

**DOI:** 10.1097/MD.0000000000014276

**Published:** 2019-01-25

**Authors:** Hongling Zhao, Qingxiong Yue, Tao Wang, Lin Wang, Zhanqi Pang, He Dong, Jian Yang, Yawen Li, Shijun Li

**Affiliations:** a Department of Neurology; b Department of Ultrasound; c Department of Cardiology, Dalian Municipal Central Hospital affiliated of Dalian Medical University, Dalian, Liaoning, China.

**Keywords:** contrast transcranial Doppler, patent foramen ovale, percutaneous closure, residual shunt, transesophageal echocardiography, transthoracic echocardiography

## Abstract

The objective of this study is to investigate the sensitivity of contrast-enhanced transthoracic echocardiography (c-TTE) versus transesophageal echocardiography (TEE) to detect a residual right-to-left shunt (RLS) following a transcatheter patent foramen ovale (PFO) closure.

A self-controlled study was conducted in a tertiary referral hospital. 57 patients with PFO who had experienced migraines underwent percutaneous PFO closure. c-TTE, TEE, and contrast-enhanced transcranial Doppler (c-TCD) at resting and Valsalva maneuver were performed during the 3-month follow-up after the closure.

The closure devices were successfully implanted in all patients without complications. Three months after closure, TEE did not detect residual Valsalva shunts in any of the 57 patients; residual valsalva shunts were found via c-TTE in 15 of the 57 patients and were also observed via c-TCD. When c-TTE and/or c-TCD were used, the rate of residual RLSs detected in patients who underwent PFO closure was 26.32%, which was significantly different than the rate detected using TEE (*P < *.05)

c-TTE and c-TCD showed equivalent sensitivity in evaluating transcatheter closure of a PFO. c-TTE could be a more cost-effective and reliable method to detect the residual shunt after PFO closure.

## Abstract 2

Supplemental Digital Content is available in the text

## 1 Introduction

A patent foramen ovale (PFO) is an intra-atrial latent conduit located between the septum primum and septum secundum that opens when the pressure of the right atrium exceeds the pressure of left atrium and leads to a right-to-left shunt (RLS). A patent foramen ovale is a common condition that occurs in up to 25% of the general population.^[1]^ An RLS caused by PFO has been implicated in the pathogenesis of migraine headaches. Some chemicals that are cleared by the lungs are shunted via a PFO and induce migraine symptoms in susceptible individuals; closure of the PFO will ameliorate or eliminate headache. Subjects with migraine could benefit from percutaneous closure of their PFO.^[2,3]^

Ultrasonography is the major method for the diagnosis of a PFO. Many studies have investigated the sensitivity of contrast-enhanced transthoracic echocardiography (c-TTE), contrast-enhanced transcranial Doppler (c-TCD), and transesophageal echocardiography (TEE) on the screening, diagnosis, and quantification of PFOs. TEE, which can show the atrial septal anatomy and an RLS, is considered to be the gold standard for the diagnosis of a PFO.^[4]^ However, TEE is a semi-invasive method that increases the discomfort of patients. c-TTE is a highly sensitive method to detect an RLS. c-TCD is also a highly sensitive method to detect an RLS and c-TCD without observing anatomy. Few studies have mentioned the sensitivity of the different ultrasonographic methods in detecting residual shunts after transcatheter closure. Therefore, the goal of this study is to investigate the sensitivity of TEE, c-TTE, and c-TCD for detecting a residual RLS following a transcatheter closure for migraine headaches, and we expect to determine the most suitable method for follow-up after a transcatheter closure.

## 2 Methods

### 2.1 Study population

Around 57 patients with PFO who experienced migraines were enrolled in this study from June 2012 to July 2016. They undergo cranio-cerebral CT or MRI in order to exclude a local cause for headaches. Subjects were excluded from the study if they had a history of seizure disorder or other organic central nervous system disease, headaches other than migraines, or evidence of alcohol or substance abuse within the previous year. Furthermore, subjects were ineligible for transcatheter closure if they had a history of intracardiac thrombus or tumour, acute or recent (within 6 months) myocardial infarction or unstable angina, left ventricular aneurysm, atrial fibrillation.

The baseline characteristics of patients are summarized in Table 1. The closure device was implanted with a Cardi-O-Fix PFO Occluder (Starway medical, Shenzhen. China). All the patients who underwent PFO closure received dual antiplatelet therapy (100 mg of aspirin plus 75 mg of clopidogrel per day) for 3 months, followed by single antiplatelet therapy for 6 months. This study was approved by the Ethics Committee of Dalian Municipal Central Hospital. Written informed consent was obtained from each patient. All patients underwent TEE with color Doppler flow imaging (CDFI), c-TTE and c-TCD performed by 3 different technicians before and 3 months after the closure procedure. The ultrasound technicians were blind to the subjects’ groups and the results of the parallel ultrasound that was performed by the other technician.

**Table 1:**
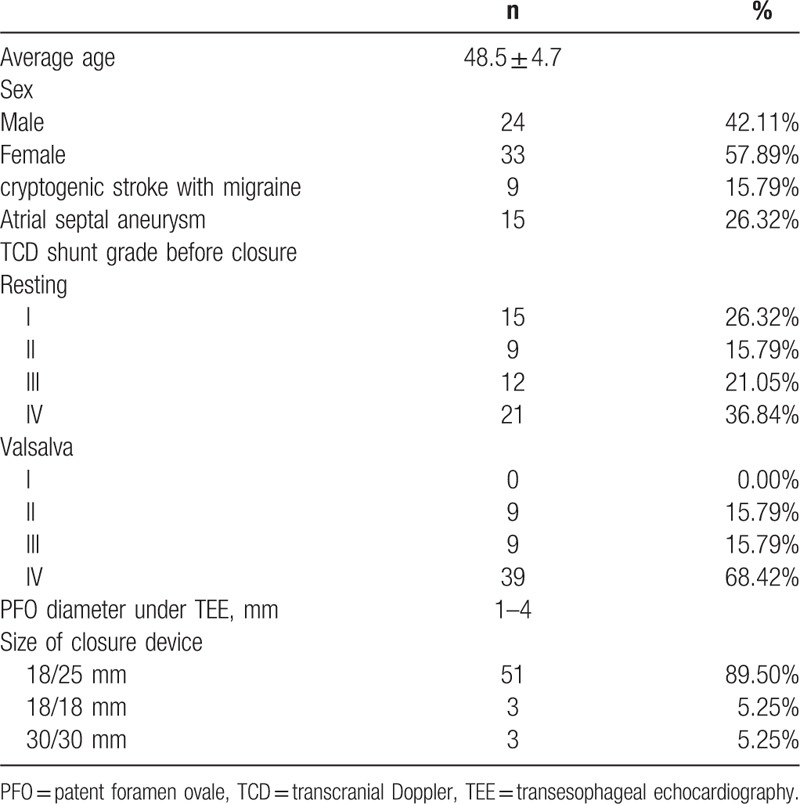
Baseline characteristics of patients with patent foramen ovale.

### 2.2 Agitated saline contrast test

A mixture of 9 mL of saline and 1 mL of air was agitated using 2 syringes connected to a 3-way stopcock to make the air–saline mix. The bolus of saline mixture was injected into the antecubital vein within 2–3 minutes.

Ultrasound (SSH-880CV (Aplio Artida), Toshiba, Japan) Criteria

**Figure 1:**
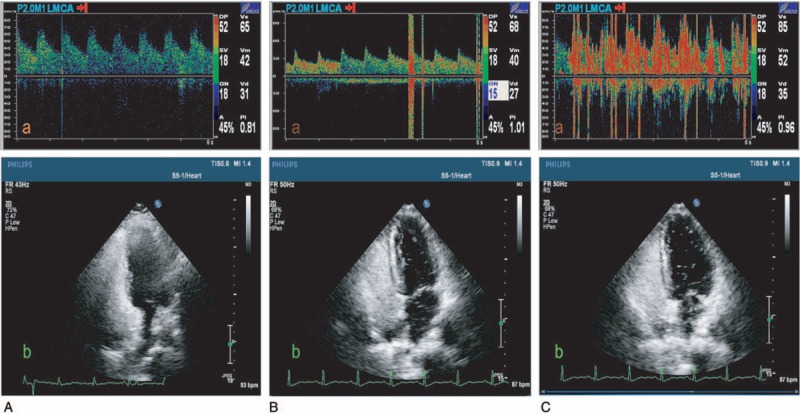
Shunt in c-TTE and c-TCD: The shunt was defined as Grade I [no microembolic signal c-TCD (Aa) and c-TTE (1Ab)], Grade II (small; 1–10 microembolic signals c-TCD Ba and c-TTE Fig. Bb), Grade III (medium; >10 microembolic signals), or Grade IV [large; >10 microembolic signals with “curtain” c-TCD (Ca) and c-TTE (Cb)].

Using c-TTE, the shunt was determined to be Grade I (no microembolic signal), Grade II (small; 1–10 microembolic signals), Grade III (medium; 10–20 microembolic signals), or Grade IV (large; >20 microembolic signals (Fig. 1)

TCD (EMS-9E, Delica, China) 2-MHz probe

Using c-TCD, the shunt was determined to be Grade I (no microembolic signal), Grade II (small; 1–10 microembolic signals), Grade III (medium; >10 microembolic signals), or Grade IV (large; >10 microembolic signals with “curtain”) (Fig. 1)

### 2.3 Statistical analysis

All data for continuous variables are reported as the mean ± standard deviation or the median. A chi-square test was used to compare categorical variables between groups. A Student's test or a Wilcoxon rank-sum test was used to compare continuous variables between groups. A value of *P < *.05 was considered to be statistically significant. All analyses were performed using SPSS software version 11.0 (SPSS, Chicago, IL).

## 3 Results

### 3.1 Patients’ characteristics

Around 57 consecutive patients (24 males, 33 females; 48.5 ± 4.7 years) were diagnosed PFO. Among 57 patients, 9 patients suffered both migraine headache and cryptogenic stroke (An ischemic within the previous 6 months with no identifiable cause other than a PFO), and 15 patients were concomitant atrial septal aneurysm. All the patients underwent percutaneous PFO closure for successful implantation of closure device without any complications (Table 1).

The sensitivity of different ultrasonographic methods to detect residual RLS after percutaneous PFO closure

**Figure 2:**
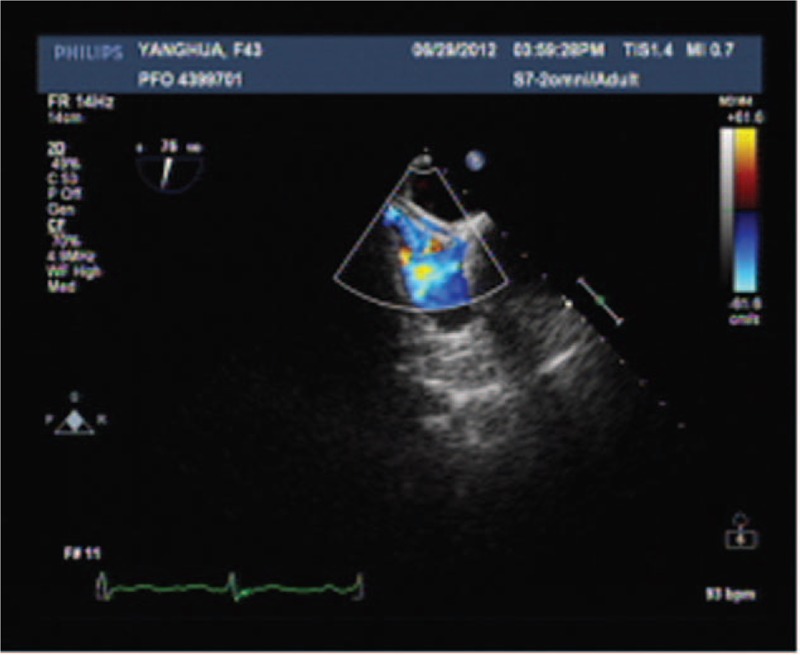
Color flow signals within closure device by TEE with CDFI, without color flow signals crossing the defect with the device in place.

Three months after undergoing a closure, patients were examined via transthoracic echocardiography with CDFI, TEE with CDFI, c-TCD and c-TTE. TEE with CDFI showed the closure device in the correct position without any signs of a residual shunt at rest or during a Valsalva maneuver. TEE with CDFI did not show any signals crossing the atrial septum or mural thrombus; Color flow signals within the closure device, but not crossing the septum were showed in 9 of the 57 patients (Fig. 2). Residual RLSs were detected in 15 of the 57 patients via c-TTE, and residual RLSs were detected in 15 of the 57 patients by c-TCD (Tables 2 and 3; supplemental digital content). When c-TTE and/or c-TCD were used, the rate of residual RLSs detected in patients who underwent PFO closure was 26.32% (Tables 2 and 3), and there was no difference between the 2 methods. TEE did not detect any residual Valsalva shunts in the 57 patients; c-TTE detected residual Valsalva shunts in 15 of the 57 patients, which were also detected by c-TCD. There was a significant difference in residual Valsalva shunt detection by c-TTE or c-TCD compared to detection by TEE (*P < *.05). Two different technicians performed the c-TTE and c-TCD were kept blind to the results of the parallel ultrasound that was performed by the other technician, and the 2 observers achieved the same result for each method. Thus, there is no inter-observer or intraobserver variability for detecting residual shunt by each method.

**Table 2:**
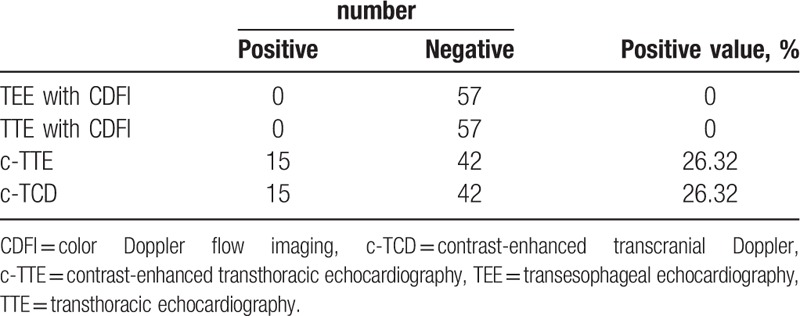
The sensitivity of different ultrasonographies on detection of residual right-to-left shunt.

**Table 3:**
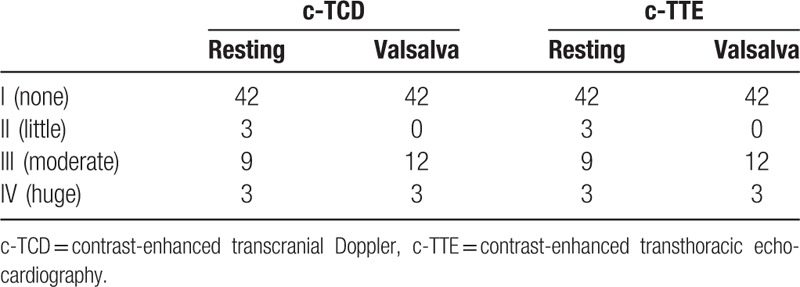
The amount of different ultrasonographies on detection of residual right-to-left shunt.

## 4 Discussion

The current study showed that TEE with CDFI did not detect residual shunts in any of the 57 patients evaluated 3 months after closure and residual shunts were detected in 15 of the 57 patients by either c-TTE or c-TCD. c-TTE and c-TCD showed equivalent sensitivity in evaluating transcatheter closure of a PFO.

Percutaneous PFO closure is a safe and effective procedure that can treat migraine headaches.^[5,6]^ However, the initial transcatheter closure still does not completely eliminate the right-to-left shunt. A residual RLS is not uncommon soon after a closure procedure, and approximately 19.5% and 18.2% of patients present with residual shunts 6 months and 12 months after closure, respectively. However, most of these residual shunts are small, with <3% being persistent large shunts 1 year after closure.^[7]^ Persistent moderate-to-severe residual shunts after closure may increase the recurrence of migraine headaches. Therefore, follow-up after a closure to detect residual shunts is of vital importance. More residual shunt **c**an be detected at 3 months after the closure procedure, thus, we chose to perform ultrasonography at 3 months after the closure procedure in order to find the most sensitive method.

Ultrasonography, including TEE, c-TTE and c-TCD, is the main method to diagnose and evaluate a PFO before and after closure. These methods have been used in different studies to evaluate residual shunts.^[7–9]^ A multicentric survey showed that 42.4% to 75% of patients who underwent a closure were evaluated by c-TTE alone, 6.5% to 23.4% of patients were fellow up with c-TCD alone or TEE alone.^[7]^ TEE and c-TTE are used in prospective, randomized, double-blinded, controlled studies to evaluate PFO, such as the RESPECT Trial,^[10]^ CLOSE Trial,^[11]^ and PRIMA Trial,^[3]^ and c-TCD was used in the PREMIUM Trial.^[12]^ Studies have shown that different ultrasonographic methods have differing sensitivities in evaluating an RLS of PFO.^[8]^ A systematic review and diagnostic test accuracy meta-analysis shows that c-TCD is more sensitive than TTE in the detection of PFO in patients with cryptogenic cerebral ischemia. The overall diagnostic yield of c-TCD appears to outweigh that of c-TTE.^[13]^ The use of c-TTE and c-TCD to detect residual shunts after percutaneous PFO closure may be more sensitive and comfortable than TEE. Our study may be helpful in the follow-up evaluations after the PFO closure.

There are several limitations in our study. First, the patient number is relative small in current study. Second, we only detect residual shunts by the 3 ultrasonographic methods and cardiac nuclear magnetic resonance (CMR) may be better method to detect residual shunts, but CMR was expensive and could not be performed for 3 months after PFO closure. Third, a receiver operating characteristic curve and calculating the the area under the receiver operating characteristic curve maybe helpful to evaluate the 3 ultrasonography methods detecting the residual shunts after percutaneous PFO closure. It is hard to make a receiver operating characteristic curve because of lacking of a golden standard, such as cardiac nuclear magnetic resonance. Only little of our patient performed TEE with agitated saline or contrast agents, and we thought it has more difficult for patients to cooperate on contrast enhanced TEE with Valsalva maneuver than contrast-enhanced TTE. We will do further studies to try to address all of the above issues.

In conclusion, this study showed c-TTE and c-TCD show equivalent sensitivity in evaluating transcatheter closure of a PFO. c-TTE could be a more cost-effective and reliable method to detect the residual shunt after PFO closure.

## Author contributions

**Investigation:** Qingxiong Yue, Jian Yang.

**Methodology:** Qingxiong Yue, Lin Wang, Zhan-qi Pang, He Dong, Ya-wen Li.

**Project administration:** Shijun Li.

**Resources:** Jian Yang, He Dong.

**Software:** Zhan-qi Pang.

**Writing – original draft:** Hong-ling Zhao, Tao Wang.

**Writing – review & editing:** Tao Wang, Shijun Li.

## Supplementary Material

Supplemental Digital Content

## Supplementary Material

Supplemental Digital Content
